# Microstructural asymmetry of the corticospinal tracts predicts right–left differences in circle drawing skill in right-handed adolescents

**DOI:** 10.1007/s00429-015-1178-5

**Published:** 2016-01-11

**Authors:** Steffen Angstmann, Kathrine Skak Madsen, Arnold Skimminge, Terry L. Jernigan, William F. C. Baaré, Hartwig Roman Siebner

**Affiliations:** 1 Danish Research Centre for Magnetic Resonance, Centre for Functional and Diagnostic Imaging and Research, Copenhagen University Hospital Hvidovre, 2650 Hvidovre, Denmark; 2 Center for Human Development, University of California, San Diego, CA USA; 3 Department of Neurology, Copenhagen University Hospital Bispebjerg, Bispebjerg, Denmark

**Keywords:** Manual dexterity, Brain development, Circle drawing, Corticospinal tract, Hemispheric asymmetry, Diffusion tensor imaging

## Abstract

Most humans show a strong preference to use their right hand, but strong preference for the right hand does not necessarily imply a strong right–left asymmetry in manual proficiency (i.e., dexterity). Here we tested the hypothesis that intra-individual asymmetry of manual proficiency would be reflected in microstructural differences between the right and left corticospinal tract (CST) in a cohort of 52 right-handed typically-developing adolescents (11–16 years). Participants were asked to fluently draw superimposed circles with their right dominant and left non-dominant hand. Temporal regularity of circle drawing movements was assessed for each hand using a digitizing tablet. Although all participants were right-handed, there was substantial inter-individual variation regarding the relative right-hand advantage for fluent circle drawing. All subjects underwent whole-brain diffusion tensor imaging at 3 Tesla. The right and left CST were defined as regions-of-interest and mean fractional anisotropy (FA) and diffusivity values were calculated for right and left CST. On average, mean FA values were higher in the left CST relative to right CST. The degree of right–left FA asymmetry showed a linear relationship with right–left asymmetry in fluent circle drawing after correction for age and gender. The higher the mean FA values were in the left dominant CST relative to the right non-dominant CST, the stronger was the relative right-hand advantage for regular circle drawing. These findings show that right–left differences in manual proficiency are highly variable in right-handed adolescents and that this variation is associated with a right-left microstructural asymmetry of the CST.

## Introduction

Humans have extraordinary ability to perform skilled and well-coordinated hand movements, which enables them to solve demanding manual tasks. This often involves the manipulation of an object, such as in writing or drawing. The high level of dexterity in humans and non-human primates has been linked to the existence of fast-conducting monosynaptic connections in the pyramidal tract that link the motor cortex to the spinal motor neurons supplying the hand muscles (Lemon 1999). These monosynaptic corticospinal projections enable independent finger movements, an ability that is fundamental to dexterity (Lemon 2008).

Another intriguing feature of manual motor control is that most humans show a clear preference towards using one hand for skilled movements. This striking example of a behavioral asymmetry is called handedness or dextrality. A series of brain imaging studies have been conducted to identify functional or structural asymmetries in the motor system that can be attributed to preferential hand use. Functional neuroimaging studies have consistently shown functional inter-hemispheric asymmetries in motor cortical regions during manual motor tasks depending on the individual degree of handedness (Dassonville et al. 1997; Volkmann et al. 1998; Kloppel et al. 2007).

At the macrostructural level, *T*_1_-weighted structural magnetic resonance imaging (MRI) has revealed an anatomical fingerprint of handedness in the human central sulcus. The central sulcus was found to be deeper in the hemisphere contralateral to the dominant hand in both right-handers and left-handers (Amunts et al. 2000). Using shape analysis, it was shown that the human hand knob, a major landmark of the hand motor representation, is located more dorsally in the left hemisphere in right-handers than in left-handers (Sun et al. 2012). These handedness-related differences in sulcus depth or shape are not captured by more traditional voxel-based methods investigating differences between left-handers and right-handers. Indeed, voxel-based morphometry (VBM) applied to *T*_1_-weighted MRI of large cohorts of healthy adults failed to reveal consistent inter-hemispheric structural asymmetries related to handedness (Good et al. 2001; Watkins et al. 2001). Another VBM study only found a statistical trend towards differences in right–left asymmetry, when comparing the asymmetry maps of young right-handed (*n* = 56) and left-handed (*n* = 56) men (Herve et al. 2006).

Other studies have used diffusion-weighted imaging (DWI) to investigate handedness related structural asymmetry in the corticospinal tract (Gong et al. 2005; Westerhausen et al. 2007; Li et al. 2010; Seizeur et al. 2013). DWI is sensitive to the diffusion of water molecules and provides information about microstructural properties of tissue, because diffusion of water molecules depends on the structural properties of encompassing tissue. In white matter, diffusion is less hindered parallel than perpendicular to the fiber bundles, because of the axonal membranes as well as the surrounding myelin sheaths and other microstructural characteristics. This explains why voxels in the white matter show tract-dependent anisotropic behavior. Diffusion properties can be characterized by applying a diffusion tensor model at the voxel level (Basser et al. 1994). Fractional anisotropy (FA) and mean diffusivity (MD) are well-established diffusion tensor-based metrics of regional water diffusion in a given voxel. The FA value indicates how much water diffusion has a directional bias, whereas the MD value describes the direction-independent overall magnitude of diffusion in the voxel (Basser et al. 1994). In addition, diffusivity can also be expressed as either parallel (λ_**║**_) or perpendicular (λ⊥) to the principal diffusion direction (Beaulieu 2009, 2002).

In children, diffusion tensor imaging (DTI) has shown age-related increases in FA and decreases in MD in multiple white matter tracts including the CST (Eluvathingal et al. 2007; Lebel et al. 2008). These age-related microstructural DTI changes provide evidence for continuing maturation of white matter tracts into early adulthood (Lebel et al. 2008). In healthy adults, DTI consistently showed inter-hemispheric asymmetries with higher FA and lower MD values in the CST of the left hemisphere. With the exception of an early DTI study (Buchel et al. 2004), handedness had no influence on the individual expression of the left > right FA asymmetry in the adult brain (Gong et al. 2005; Westerhausen et al. 2007; Li et al. 2010; Seizeur et al. 2013). Handedness is a multidimensional construct that comprises many facets and aspects (Healey et al. 1986; Janssen 2004) and hand preference tests, such as the Edinburgh Handedness Inventory (Oldfield 1971), do not consider right-left asymmetries in manual proficiency (i.e., dexterity). Therefore, previous DTI studies might have yielded negative results because they tested for an association between microstructure and handedness rather than manual dexterity. Interestingly, a recent study in a small sample of 14 adults, employing DWI tractography, observed that manual dexterity in grip strength and finger-tapping performance was associated with a left higher than right FA asymmetry of respectively the CST and intrahemispheric pre- and post central gyrus connections (Rose et al. 2012).

In the present study we used DTI to test the hypothesis that the individual expression of dexterity is represented in the microstructure of the main corticospinal pathway, which conveys cortical commands to the hand. We tested this hypothesis in typically-developing right-handed adolescents because CST maturation as indexed by DTI measures is still ongoing in this period of life. We only included dextral individuals with strong preference for the right hand because we were interested to identify microstructural asymmetry in right and left CST related to motor proficiency beyond preferred hand use. To this end, participants were asked to perform a circle-drawing task with the right and left hand, respectively, while drawing movements were recorded with a digitizing tablet (Henkel et al. 2001; Siebner et al. 2002). A kinematic measure of unimanual proficiency would better reflect right–left asymmetry in manual ability than handedness questionnaires, which capture differences in preferred hand use, but not skill, across a range of motor tasks (Rose et al. 2012).

## Materials and methods

### Subjects

Sixty-five typically-developing right-handed children and adolescents from three schools in the Copenhagen suburban area participated in the study. All participants were enrolled in a longitudinal study to prospectively trace developmental changes, called the “HUBU” project (“HUBU” = “*Hjernens Udvikling hos Børn og Unge”—*Brain maturation in children and adolescents). Participants were examined every 6 months, both behaviorally and in the MR scanner. Data used in the present study were collected in the eighth assessment round of the HUBU project. A detailed description of the HUBU cohort is given elsewhere (Madsen et al. 2010, 2011; Vestergaard et al. 2011; Klarborg et al. 2013). Exclusion criteria were any known history of neurological or psychiatric disorders or significant brain injury according to parent reports. According to the Edinburgh Handedness Inventory (Oldfield 1971), participants were consistently right-handed with handedness scores ranging from 44 to 100 (median score: 100). Prior to participation and after receiving oral and written explanation of the study aims and study procedures, all children assented to the procedures and informed written consent was obtained from the parents or guardians of all subjects. The study was approved by the local Danish Committee for Biomedical Research Ethics (H-KF-01-131/03) and conducted in accordance with the Declaration of Helsinki.

Of the 65 right-handed individuals who participated in the eighth assessment, 13 subjects were excluded because of the following reasons: no MRI acquired due to dental braces (*n* = 8), poor MR-image quality (*n* = 2), technical problems with the behavioral task (*n* = 1), incidental findings on MRI (*n* = 1) and parent reported psychiatric disorder (*n* = 1). Thus, the final sample consisted of 52 right-handed individuals (18 males and 34 females). The mean age was 13.7 years (standard deviation = 1.75, range: 11–16 years).

### Circle drawing task

**Fig. 1:**
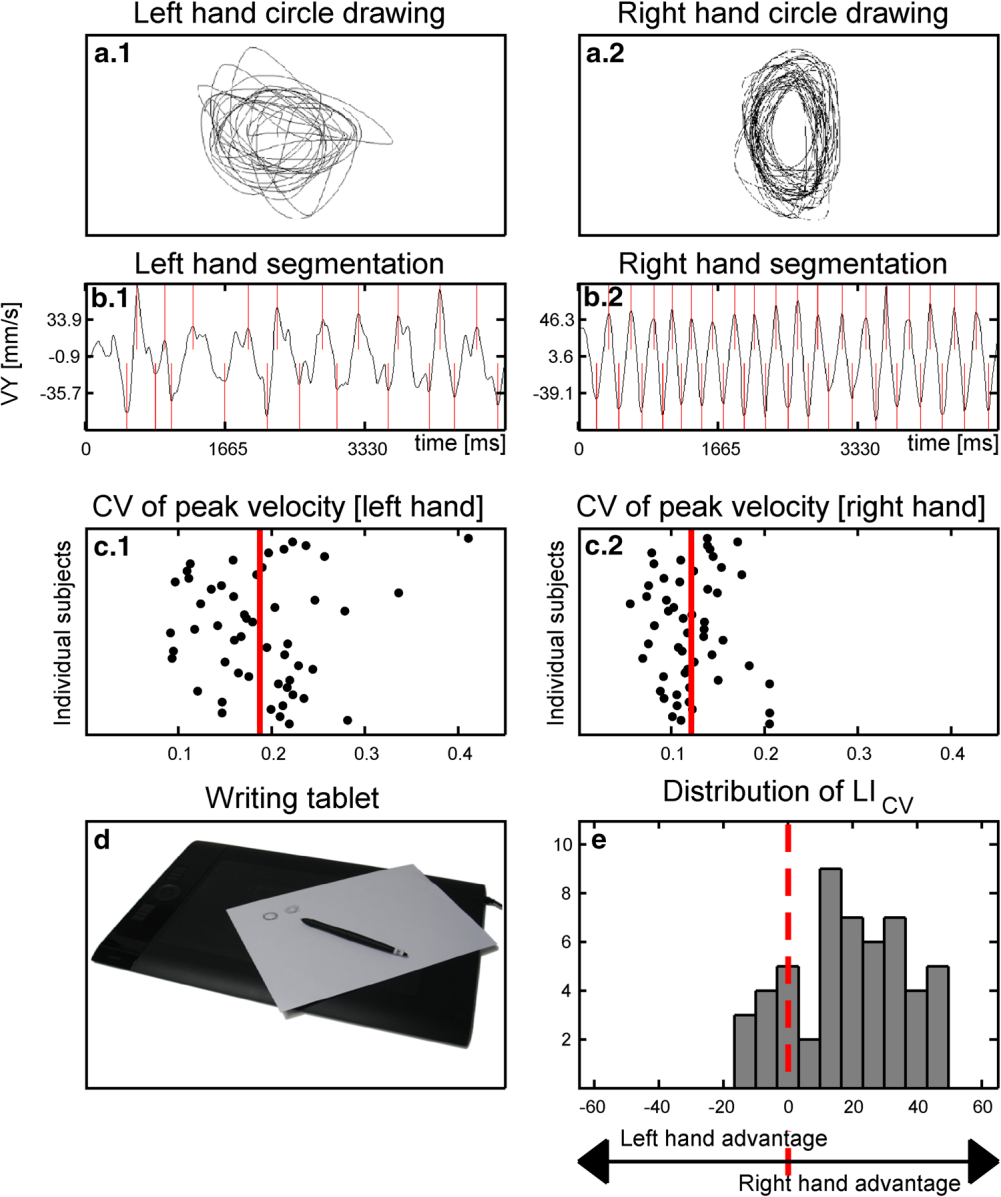
Recording of *circle* drawing and calculation of the laterality indices (LI_CV_). **a** Subjects were required to draw concentric superimposed circles on a digitizing tablet with their *right* dominant or *left* non-dominant hand. **b** *Left* and *right* hand traces were segmented into sequential up- and down-strokes and **c** the coefficient of variance (CV) of velocity stroke peaks (CV) was calculated separately for *circle* drawing movements with the *right* or *left* hand. **d** A laterality index (LI_CV_) was calculated based on the mean CV of the *right* and *left* hand CV. **e** Histogram depicting the distribution of the LI_CV_ in the 52 participants (age range: 11–16 years)

Subjects performed a simple circle drawing task with their right and left hands (Fig. 1a). Participants performed the circle drawing task with the ulnar part of the hand resting on the table. This ensured that circle drawing only involved coordinated movements of the hand and finger, while the forearm maintained a constant position. Participants were asked to continuously draw concentric superimposed circles at a convenient speed. They were informed that we were mainly interested in testing how well they can produce circles in a regular fashion. Therefore, we emphasized that they should try to smoothly produce consecutive circles at a constant speed. To facilitate fluent open-loop performance, participants were told that it was not necessary to ensure that one circle exactly overlay the other. First, they drew circles with their right dominant hand in clockwise direction for 10 s. Thereafter, participants drew circles with their left hand in counter-clockwise direction for 10 s, producing the homologous movement pattern as in the right-hand task. Because of the simplicity of the task, no test runs were performed.

A pressure sensitive digitizing tablet (WACOM Intuos4 large PTK-840, Wacom Technology Corporation, Vancouver, WA, USA) recorded the writing trace that was produced by the tip of the pen at a sample rate of 200 Hz. Kinematics of the writing movements were analyzed with commercial software (CS-Win, version 10.05, MedCom, Munich, Germany).

### Kinematic analysis of circle drawing

The vertical drawing traces and the corresponding velocity profiles produced with the right and left hand were automatically segmented into consecutive up-and-down strokes. Curves of vertical velocity were calculated and smoothed by nonparametric regression methods (Fig. 1b), (Marquardt and Mai 1994). We were mainly interested in assessing the temporal regularity of drawing movements. Therefore, we chose the variability of peak vertical velocity during circle drawing as the kinematic measure of interest and calculated the coefficient of variance (CV = standard deviation/mean) for peak drawing velocity. The higher the CV, the less proficient were the circle drawing movements.

The CV of peak vertical drawing velocity was calculated separately for right hand drawing (CV_R_) and left hand drawing (CV_L_). We then calculated a laterality index (LI_CV_) as an asymmetry measure for manual proficiency, using the following formula.

1 $${\text{LI}}_{\text{CV}} = \frac{{{\text{CV}}_{\text{R}} \, - \,{\text{CV}}_{\text{L}} }}{{{\text{CV}}_{\text{R}} \, + \,{\text{CV}}_{\text{L}} }} \times ( - 100)$$

Values between −100 and −1 revealed a higher proficiency of circle drawing for the left hand which produced more regular velocity profiles than the right hand. Conversely, values between 1 and 100 indicate a right-hand advantage for regular circle drawing. Figure 1 shows the parameterization of the LI_CV_.

### Magnetic resonance imaging

All subjects were scanned using a 3T Siemens Magnetom Trio MR scanner (Siemens, Erlangen, Germany) with an eight-channel head coil (Invivo, Fl, USA). All acquired scans were aligned parallel to the anterior-posterior commissural line. High-resolution 3D *T*_1_-weighted images of the whole head were acquired using a MPRAGE sequence (TR = 1550 ms, TE = 3.04 ms, matrix 256 × 256, 192 sagittal slices with no gap, 1 mm^3^ isotropic voxels). Whole brain diffusion-weighted (DW) images were acquired using a twice-refocused balanced spin echo sequence that minimized eddy current distortion (Reese et al. 2003). Ten non-DW images (*b* = 0) and 61 DW images (*b* = 1200 s/mm^2^), encoded along independent collinear diffusion gradient orientations, were acquired (TR = 8200 ms, TE = 100 ms, FOV = 220 × 220, matrix = 96 × 96, GRAPPA: factor = 2, 48 lines, 61 transverse slices with no gap, 2.3 mm^3^ voxels). A gradient echo field map was acquired to correct b0 field distortions (TR = 530 ms, TE[1] = 5.19 ms and TE[2] = 7.65 ms, FOV = 256 × 256; matrix = 128 × 128, 47 transverse slices with no gap, voxel size = 2 × 2 × 3 mm^3^).

### Image pre-processing and analysis

Prior to analysis and blind to behavioral data, raw images from all subjects were visually inspected to ascertain sufficient image quality. Images were preprocessed using pipelines implemented in MATLAB, using mainly SPM 8 (Wellcome Department of Cognitive Neurology, University College London, UK) routines. *T*_1_-weighted images were corrected for spatial distortions due to non-linearity in the gradient system of the scanner (Jovicich et al. 2006) and oriented to the MNI coordinate system using a six-parameter mutual information rigid transformation. Subsequently, the mean b0 image was co-registered to the *T*_1_-weighted image using a six-parameter mutual information rigid transformation after which all diffusion-weighted images were co-registered (no reslicing) to the mean b0 image. Next, all co-registered images were corrected for geometric distortions using a voxel displacement map based on both the acquired b0 field map (Andersson et al. 2001) and the scanner-specific gradient non-linearity (Jovicich et al. 2006). Finally, all images were resliced into the MNI coordinate system using tri-linear interpolation. Note that this procedure involves only one re-slicing step. The diffusion gradient orientations were adjusted to account for any rotation applied during registration. The diffusion tensor was fitted using a least-squares-fit by non-linear optimization employing a Levenburg-Marquardt algorithm and constrained to be positive definite by fitting its Cholesky decomposition as embedded in Camino. Fractional anisotropy (FA), mean diffusivity [MD = (*λ*_1_ + *λ*_2_ + *λ*_3_)/3] as well as diffusivity parallel (λ_║_ = *λ*_1_) and perpendicular [λ⊥ = (*λ*_2_ + *λ*_3_)/2] to the principal diffusion direction were calculated. A brain mask based on the realigned and distortion corrected b0 images was applied to the FA and diffusivity images.

### Inter-subject spatial normalization of fiber tracts

All DTI measures of interest (i.e., FA, MD, λ_**║**_ and λ⊥) were extracted from regions-of-interest (ROIs) to test specific hypotheses and to determine the anatomical specificity of observed associations (see below). Spatial normalization and alignment of fiber tracts across subjects was achieved using the Tract-Based Spatial Statistics (TBSS) module, part of FSL 4.1.4 (Smith et al. 2006), which was used to project DTI measures onto a mean FA skeleton that represents the centers of all tracts common to the group. As we were interested in investigating right–left difference in CST microstructure, we generated a symmetric skeleton.

At first, subjects’ FA images were aligned into a common space using the non-linear registration tool FNIRT (Andersson et al. 2007). A study-specific target, the groups’ most representative FA image, was then identified after non-linearly registering each subjects FA image to every other subjects FA image. Next, the target FA image was aligned to MNI space using affine registration and subsequently the entire aligned dataset was transformed into 1 mm^3^ MNI space. A cross-subject mean FA image was created and thinned to create a mean FA skeleton, representing the centers of all tracts common to the group. The mean FA skeleton was thresholded at FA > 0.25 and contained 134.184 mm^3^ interpolated voxels, corresponding to approximately one quarter of the voxels with FA above 0.25. The aligned FA images of each subject were then projected onto the mean skeleton by locating the highest local FA value in the direction perpendicular to the skeleton tracts and assigning this value to the skeleton. Next we generated the symmetric skeleton. First, a symmetric mean FA map was generated by averaging the mean FA map and a (left–right) flipped version of the mean FA image. The symmetric mean FA map was thinned, and masked with a (one voxel) dilated version of the original mean FA skeleton, ensuring that only skeleton segments that were almost symmetric in the original non-symmetric skeleton were included. Finally, the preliminary symmetric skeleton was masked with its left–right flipped counterpart to generate a symmetric mean skeleton, which contained 112.616 voxels. In addition, the non-linear warps, skeleton projections and symmetric skeleton generation were applied to the MD, λ_**║**_ and λ_⊥_ data.

### Regions-of-interest (ROIs)

**Fig. 2:**
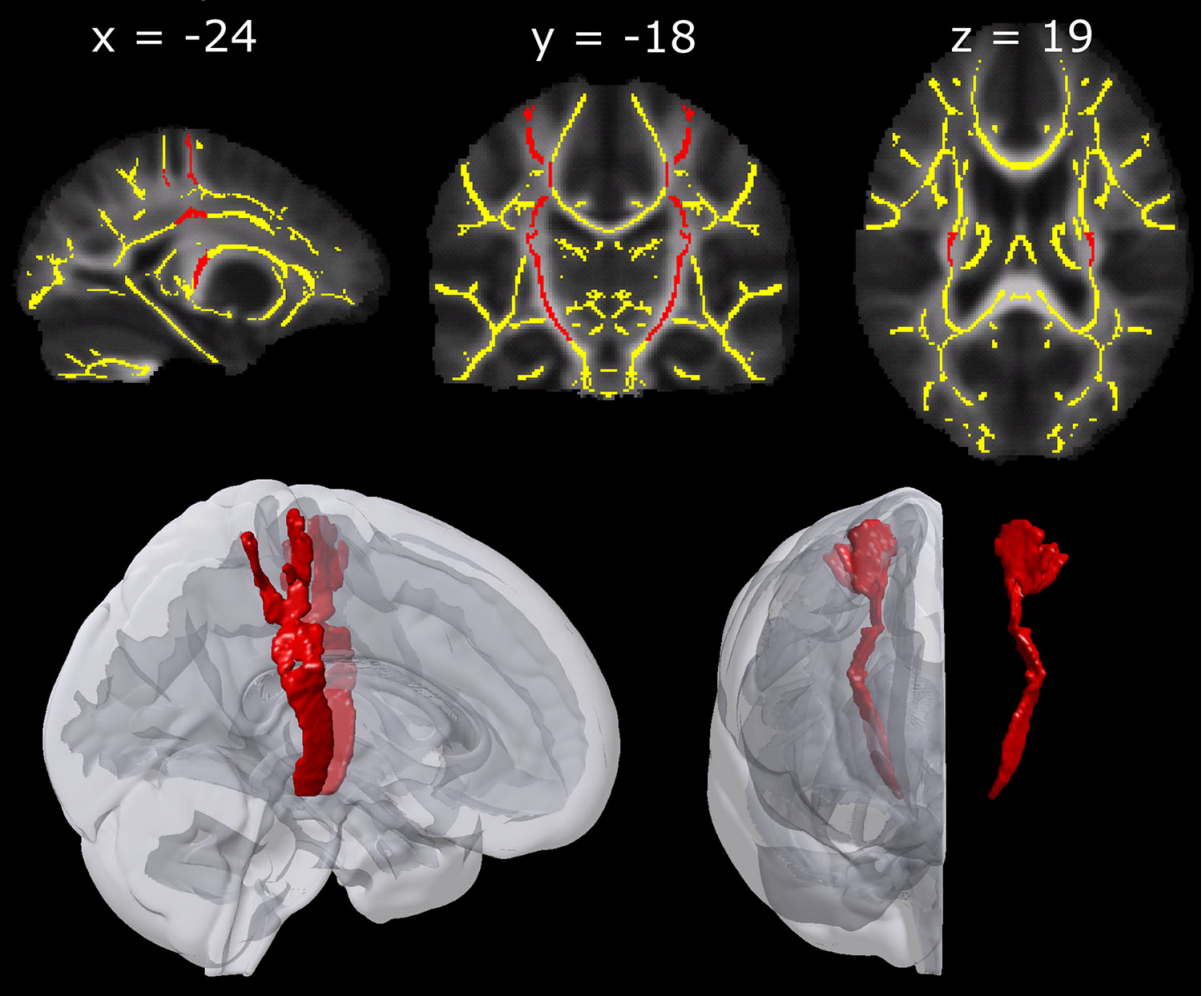
The *upper* panels show the ROIs of the corticospinal tract (marked in *red*) and the symmetric TBSS skeleton (marked in *yellow*). The ROIs and the skeleton are overlaid on corresponding axial, coronal and sagittal slices of a group map displaying mean FA values in stereotactic space. The *lower* panel depicts a smoothed 3D visualization of the ROIs and their position within the standard MNI-152 template

ROIs were drawn onto the symmetric skeleton overlaid on a symmetric mean FA image in FSLview. The primary motor and sensory hand area was taken as the cortical origin for the CST containing the corticospinal connections supplying the hand muscles. This was in accordance with findings by (Kumar et al. 2009) who demonstrated in a sample of adolescents that the CST originated both from pre- and postcentral gyri. The hand knob in the motor cortex was identified (Yousry et al. 1997) and the white matter underlying the hand knob and the white matter underlying the corresponding region in the sensorimotor cortex was included in our CST ROIs. Below the level of the corpus callosum, information from the probabilistic fiber atlas implemented in FSLview (Hua et al. 2008) was used to delineate the corticospinal tract ROIs down through the posterior limb of the internal capsule. Manual fine-tuning of the ROIs was guided by the superior-inferior direction (blue) in the target’s color-coded FA map and a white matter atlas (Mori et al. 2005). No voxels were included inferior to MNI slice *z* = −14. Figure 2 shows the ROIs of the CST. Each ROI contained 1.785 voxels.

FA data from both the left and the right hemisphere were extracted separately. As for the kinematic data, we calculated a laterality index for the FA data (LI_FA_) using the formula.

2 $${\text{LI}}_{\text{FA}} = \frac{{\sum\nolimits_{{{\text{CST}}_{\text{R}} }} {\text{FA}} - \sum\nolimits_{{{\text{CST}}_{\text{L}} }} {\text{FA}} }}{{\sum\nolimits_{{{\text{CST}}_{\text{R}} }} {\text{FA}} + \sum\nolimits_{{{\text{CST}}_{\text{L}} }} {\text{FA}} }} \times 100$$

Values between -100 and -1 reflect higher FA values in the left CST, while values between 1 and 100 indicate higher FA values in the right CST. LIs were also calculated for MD, λ_**║**_ and λ_⊥_. To be able to control for global hemispheric left–right asymmetries we calculated the same asymmetry index for all skeleton voxels in the left vs. the right hemisphere.

### Statistical analysis and visualization

Statistical analyses were performed using SPSS software (SPSS 19, IBM, Somers, NY, USA). Independent and dependent variables did not significantly deviate from the Gaussian distribution as ascertained with the Shapiro–Wilk test. Effects of age and gender on circle-drawing performance, FA and MD values were examined using linear regressions. Laterality in the behavioral and ROI measures was examined using multiple linear regression models controlling for age and gender.

All assumptions for linear regressions were met and multiple linear regression tests were performed hierarchically. Our main hypotheses were that individual differences in the degree of laterality of drawing stability (i.e. laterality index LI_CV_) would be associated with the degree of laterality in CST FA and MD, after adjusting for age and gender. These hypotheses were tested employing a two-sided statistical threshold of 0.025 (Bonferroni corrected for two tests as we tested for two diffusion parameters).

Planned follow-up analyses were contingent on observing significant associations with FA and MD in our primary analyses. First, to assess anatomical specificity of observed associations, we additionally factored whole hemisphere white matter asymmetry as a nuisance regressor into the analyses. Second, to assess the relative contribution of the left and right CST, we modeled left and right CST FA both as simultaneous and as separate predictors of LI_CV_, after adjusting for age and gender. To further explore the nature of observed FA effects, we applied the same analysis to λ_**║**_ and λ⊥, as these measures may give additional information about the underlying microstructural indices. Follow-up analyses were considered significant when *p* ≤ 0.05.

To obtain further anatomical information about the lateralization of the CST and its relation to behavioral asymmetry on a smaller spatial scale, we performed two additional steps of analyses. We calculated the laterality indices for each axial slice (LI_FA-slice_) in each subject without correction for gender and age to identify topographical variations in the expression of right-left differences along the CST. Slice-wise calculations were performed for each of the 85 axial slices along the *z*-axis of MNI stereotactic space, from slice *z* = −14 to *z* = 70. Based on these 85 LI_FA-slice_-indices of our 52 participants, a one-sample permutation test was performed (100.000 permutations) in MATLAB to test which parts of the CST showed significant asymmetry in FA values. The tests were done using an approach originally developed for EEG-data (Blair and Karniski 1993) and implemented in MATLAB (Groppe et al. 2011). This approach relies on point-wise permutations of series of data and a *t*_max_ statistic. We employed an overall family-wise error rate of *α* = 0.05. Moreover, we calculated uncorrected slice-wise correlations between LI_FA-slice_ and LI_CV_ in order to provide complementary information about the regional distribution of the association between the degree of FA-laterality and the degree of behavioral laterality throughout the course of the CST ROI. For this exploratory follow-up analysis, no correction for gender and age was performed.

In addition, we performed a complementary voxel-based analysis on the whole white matter skeleton of the association between the right-left voxel-wise difference and LI_CV_, controlling for age and gender to test whether our findings were specific for the CST or whether other brain regions also showed similar effects. To do so, we employed the randomize module in FSL (Nichols and Holmes 2002) using 10.000 permutations. Significant associations were determined using the threshold-free cluster enhancement method (TFCE) (Smith and Nichols 2009) optimized for 2D-like white matter skeletons with a corrected threshold of *p* < 0.05. Finally, an effect-size map is presented to provide further anatomical information about the effects outside the CST. The effect-size map is a *t*-map of the association between the right–left voxel-wise difference and LI_CV_, controlling for age and gender.

## Results

### Circle drawing

**Fig. 3:**
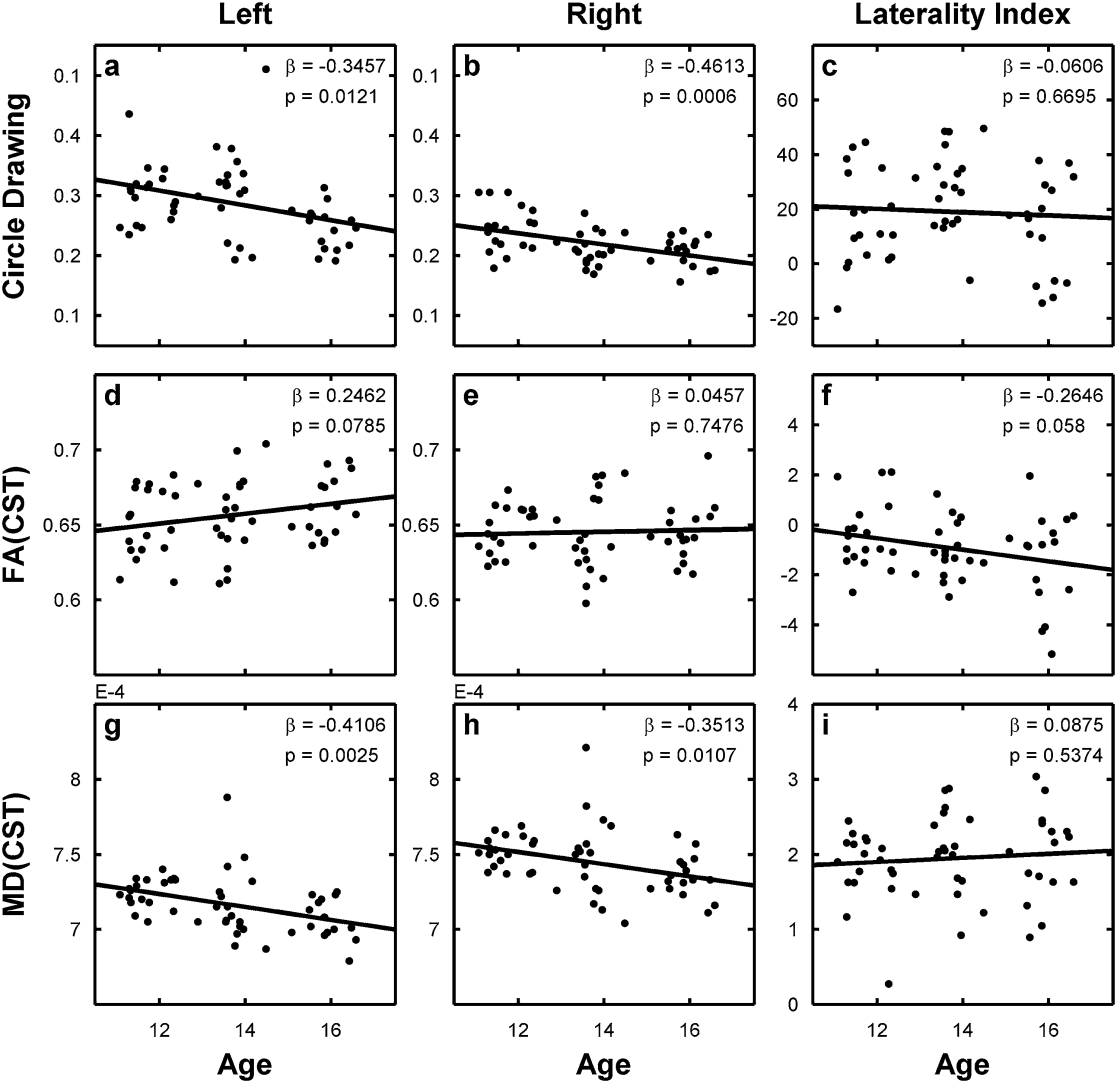
Scatter plots showing the linear relationship between *circle* drawing (**a**–**c**), FA values (**d**–**f**,) and MD values (**g**–**i**) and age of the participants. Regressions are calculated separately for *left* (**a**, **d**, **g**), *right* (**b**, **e**, **h**) and laterality indices (**c**, **f**, **i**). Data are linearly interpolated with a *black line* and regression coefficient (*β*) and significance level (*p*) are given in the *upper right* corner of the respective plot

Most participants showed a right-hand advantage for regular circle drawing, producing less variable peak velocities with their right dominant hand than with the left non-dominant hand (Fig. 3c). Although all participants were consistent right-handers, the relative right-hand advantage for fluent circle drawing varied considerably across participants. Some subjects showed no clear difference in CV of peak velocity between the right and left hand or even a slight left-hand advantage, whereas others showed a clear right-hand advantage (Fig. 1e).

Analyses revealed that variability of circle drawing velocity linearly decreased with age (Fig. 3a, b). This age-dependent improvement in temporal stability of circle drawing was comparable for the right dominant and left non-dominant hand. Accordingly, the laterality index LI_CV_ reflecting the right-left asymmetry of circle drawing performance did not change as a function of age (Fig. 3c). We found no gender effects on LI_CV_ (*β* = 0.074, *p* = 0.602). There was a statistical trend towards a significant interaction between age and gender for left-hand circle drawing (*β* = −0.26, *p* = 0.062) and the circle drawing laterality index (*β* = −0.264, *p* = 0.059), caused by a trend towards an earlier improvement in temporal regularity of left-hand circle drawing with age in boys relative to girls.

### Microstructural asymmetry of the corticospinal tract (CST)

In the majority of our right-handed participants, mean FA values in the left CST were higher than in the right CST (Fig. 3f), resulting in a negative right-left laterality index for FA (LI_FA_). Yet, intra-individual asymmetry of mean FA in the CST varied substantially across participants, and more than 20 % of the participants had a positive LI_FA_. There was a trend towards a linear increase of FA values with age in the left CST (*β* = 0.246, *p* = 0.079; Fig. 3d), but not in the right CST (*β* = 0.046, *p* = 0.748; Fig. 3e). Accordingly, the LI_FA_ also tended to become more negative with age (*β* = −0.265, *p* = 0.058; Fig. 3f). Mean FA in right CST was higher in males than females (*β* = −0.28, *p* = 0.044). This was not the case for left CST (*b* = −0.17, *p* = 0.22).

Mean MD in the left and right CST showed a significant linear decrease with age (Fig. 3g, h). Lower MD values were observed in the left relative to the right CST, but the left–right relation remained unchanged across the age range (*β* = 0.09, *p* = 0.537, Fig. 3i). None of the DTI-based asymmetry indices showed any significant effect for gender (all *p* values > 0.25) or any age by gender interaction (all *p* values > 0.13).

### Relation between asymmetry in dexterity and CST microstructure

**Fig. 4:**
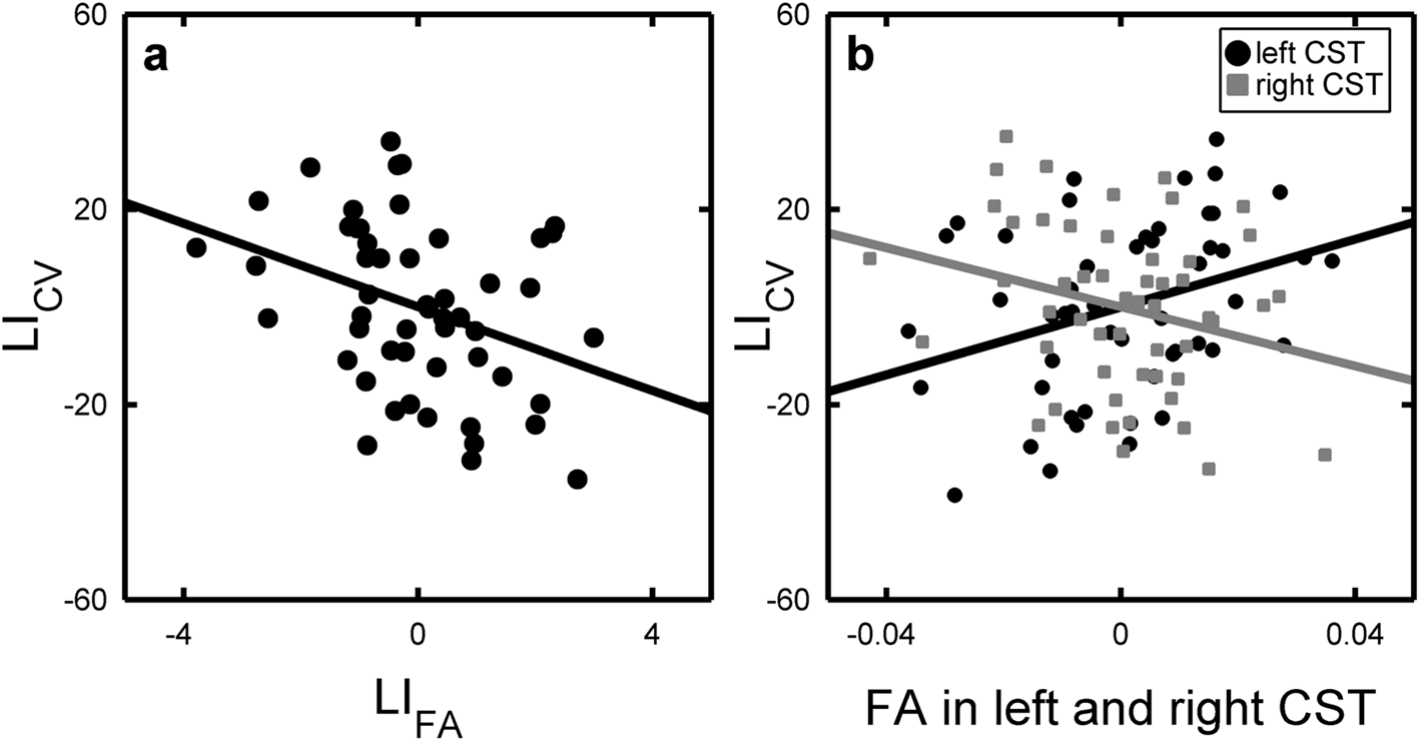
Partial regression plots predicting behavioral asymmetry (LI_CV_) as a function of corticospinal tract asymmetry (LI_FA_) in figure (**a)** and *left* and *right* FA values simultaneously but as separate regressors (**b**). Regressions are adjusted for age and gender

The multiple regression model confirmed our primary hypothesis that the asymmetry in mean FA between the right and left CST (LI_FA_) was a significant positive predictor of right–left asymmetry in drawing performance as indexed by LI_CV_ (Table 1, model 1 and Fig. 4a). The stronger the right < left FA asymmetry in CST (i.e., the lower LI_FA_), the stronger was the relative right-hand advantage for fluent circle drawing (i.e., the higher was the LI_CV_). Adjusting for hemispheric asymmetry in white matter FA values between right and left hemisphere did not change our results (Table 1, model 1b). Right–left asymmetry of mean MD in the CST did not predict right–left differences in drawing performance (Table 1, model 2).

**Table 1:** Linear regression models predicting circle drawing laterality (LI_cv_) with right–left asymmetry of fractional anisotropy (FA) and mean diffusivity (MD) in the ROI covering the corticospinal tract

Model	*R* ^2^	DTI measure	LI		Age		Gender		WH_LI_	
			*β*	*p*	*β*	*p*	*β*	*p*	*β*	*p*
1^a^	0.118	FA	−0.343	**0.019**	−0.154	0.278	0.047	0.730	−	−
1^b^	0.147	FA	−0.371	**0.012**	−0.127	0.372	0.041	0.762	0.175	0.218
2^a^	0.011	MD	0.020	0.892	−0.063	0.671	0.074	0.618	−	−

Significant results with *p* < 0.05 are highlighted in bold

*WH*_*LI*_ Whole hemisphere laterality index

^a^Models not controlled for whole hemisphere FA asymmetry

^b^Models controlled for whole hemisphere FA asymmetry

The results of the main analysis motivated planned follow-up analyses to further explore the nature of the effects (see Table 2). We investigated FA values in left and right CST both separately and simultaneously and applied the same analysis to λ_**║**_ and λ⊥, as these measures might give additional information about the underlying microstructural indices. When modeled as simultaneous predictors of LI_CV_, mean FA in both the right and left CST contributed significantly to the prediction of right-left differences in drawing performance (Table 2, model 3 and Fig. 4b): Mean FA values in left CST showed a positive linear relationship with LI_CV_. Conversely, mean FA values in right CST showed a negative relation with LI_CV_. In addition, diffusivity parallel (λ_**║**_) and diffusivity perpendicular (λ_⊥_) to the main axis of the diffusion tensor model exhibited significant associations with LI_CV_. Left CST λ_**║**_ was positively associated with right–left asymmetry in drawing performance, whereas right CST *λ*_**║**_ showed a negative linear relationship with LI_CV_. An opposite pattern emerged for the left CST, where left CST λ_⊥_ displayed a negative linear relationship with LI_CV_, whereas right CST λ_⊥_ showed a trend towards a positive linear relation with LI_CV_. Importantly, this was only true when the DTI-based measures were modeled simultaneously, but not separately (Table 2, *b* models). Together, the results show that it was not the microstructure of either the left or right CST alone that accounted for the left–right asymmetry in circle drawing. The results rather show that it is the relation between the microstructural properties of the right and left CSTs, which is associated with asymmetry between left and right hand circle drawing abilities.

**Table 2:** Linear regression models (follow-up models) predicting circle drawing laterality (LI_cv_) with diffusivities in the left and right corticospinal tract, both simultaneously and separately

Model	*R* ^2^	DTI measure	Left ROI		Right ROI		Age		Gender	
			*β*	*p*	*β*	*p*	*β*	*p*	*β*	*p*
3^a^	0.121	FA	0.462	**0.020**	−0.361	0.064	−0.162	0.265	0.063	0.661
4^b^	0.233	FA	0.222	0.139	−	−	−0.123	0.404	0.120	0.407
5^b^	0.113	FA	−	−	−0.058	0.702	−0.062	0.670	0.062	0.683
6^a^	0.372	λ_║_	0.484	**0.013**	−0.428	**0.037**	−0.134	0.372	0.103	0.453
7^b^	0.230	λ_║_	0.215	0.145	−	−	−0.021	0.884	0.104	0.466
8^b^	0.126	λ_║_	−	−	−0.086	0.587	−0.099	0.529	0.071	0.623
9^a^	0.319	λ_⊥_	−0.542	**0.034**	0.459	0.066	−0.147	0.326	0.067	0.643
10^b^	0.184	λ_⊥_	−0.169	0.279	−	−	−0.124	0.418	0.114	0.437
11^b^	0.104	λ_⊥_	−	−	0.037	0.813	−0.057	0.702	0.068	0.653

Significant results with *p* < 0.05 are highlighted in bold

^a^Models including the left and right ROI simultaneously as predictors of LI_CV_, controlling for age and gender

^b^Models including the left and right ROI as separate predictors of LI_CV_, controlling for age and gender

### Slice-wise expression of structural right–left asymmetry

**Fig. 5:**
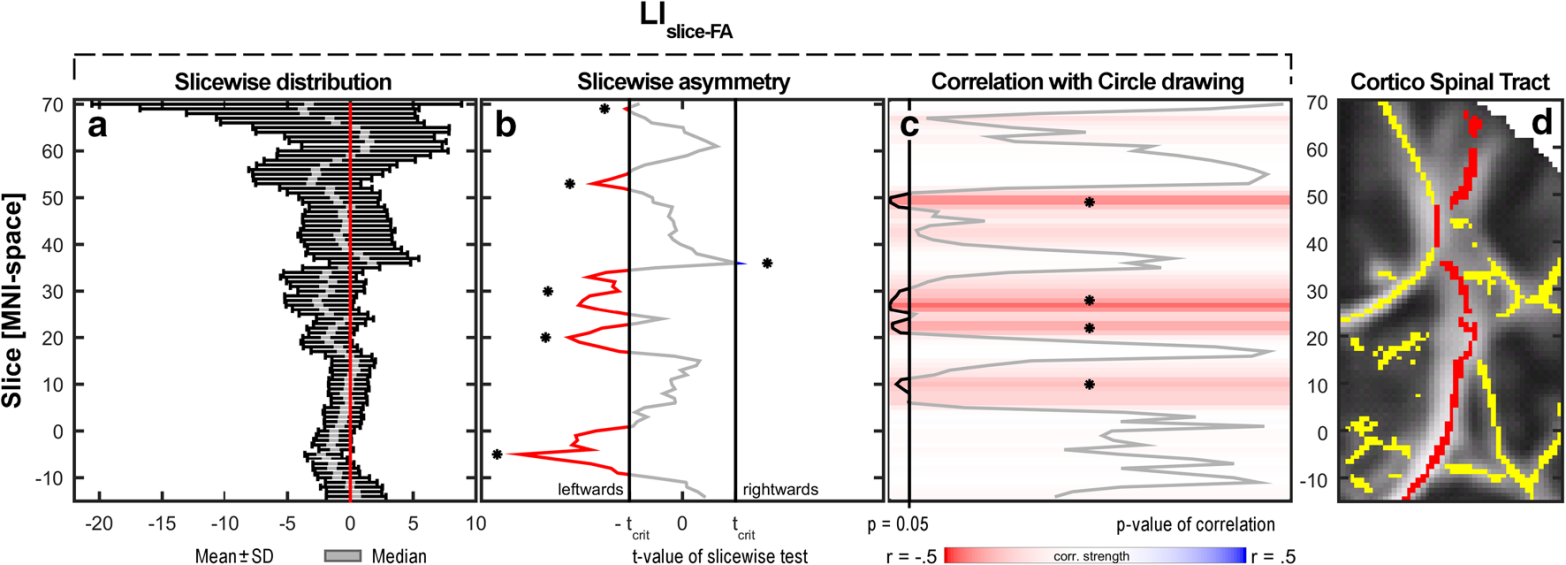
Slice-wise expression of the right–left asymmetry in mean FA (LI_FA-slice_) in axial slices of the corticospinal tract from *z* = −14 to *z* = 70 in MNI-space. **a** Distribution of LI_FA-slice_ values in the whole sample. For each slice are shown means and standard deviations as *black bars* overlaid by the median in *grey*. **b** Slice-wise asymmetry as assessed with a permutation test (corrected for multiple comparisons). The *red-grey-blue line* indicates *t*-values. The *vertical black lines* indicate corrected, two-tailed threshold criteria of significance; *t*-values not contained between these are significantly different from zero, i.e. asymmetric, and marked in *red* (*leftwards*) or *blue* (*rightwards*). **c** Slice-wise correlations (uncorrected for age and gender) with the writing laterality index LI_CV_. Color of the slice indicates correlation strength (*r*-value); *black line* indicates *p* value of the correlation test. **d** Partial coronal slice (at *y* = −18) showing the skeleton (*yellow*) overlaid on the mean FA map from *z* = −14 to *z* = 70. The *red color* indicates the part of the skeleton included in the CST-ROI

Slice-wise spatial expression of right < left asymmetry in FA varied considerably along the CST (Fig. 5). Permutation analysis revealed several clusters of slices where the right < left asymmetry in mean FA reached significance at the group level, whereas such asymmetry did not reach significance in other portions of the CST (Fig. 5b). The slice-wise expression of the linear relationship between right–left asymmetry in FA (LI_FA-slice_) and drawing skill (LI_CV_) also varied substantially along the CST (Fig. 5c). The CST sections showing the strongest association between LI_FA-slice_ and LI_CV_ were clustered around the axial slices at *z* = 10 (corresponding to the internal capsule), *z* = 27 (corresponding to the superior corona radiata), and slice *z* = 49, which is a complex region containing fibers from different tracts, e.g. corpus callosum, CST and superior longitudinal fasciculus. Of note, the slices showing the strongest right < left asymmetry in FA values were not necessarily those slices, where the degree of right < left asymmetry predicted a right-hand advantage for circle drawing. Figure 5d depicts the position of the ROI in the MNI *z*-slices −14 to 70.

### Voxel-wise analysis of the entire white-matter skeleton

**Fig. 6:**
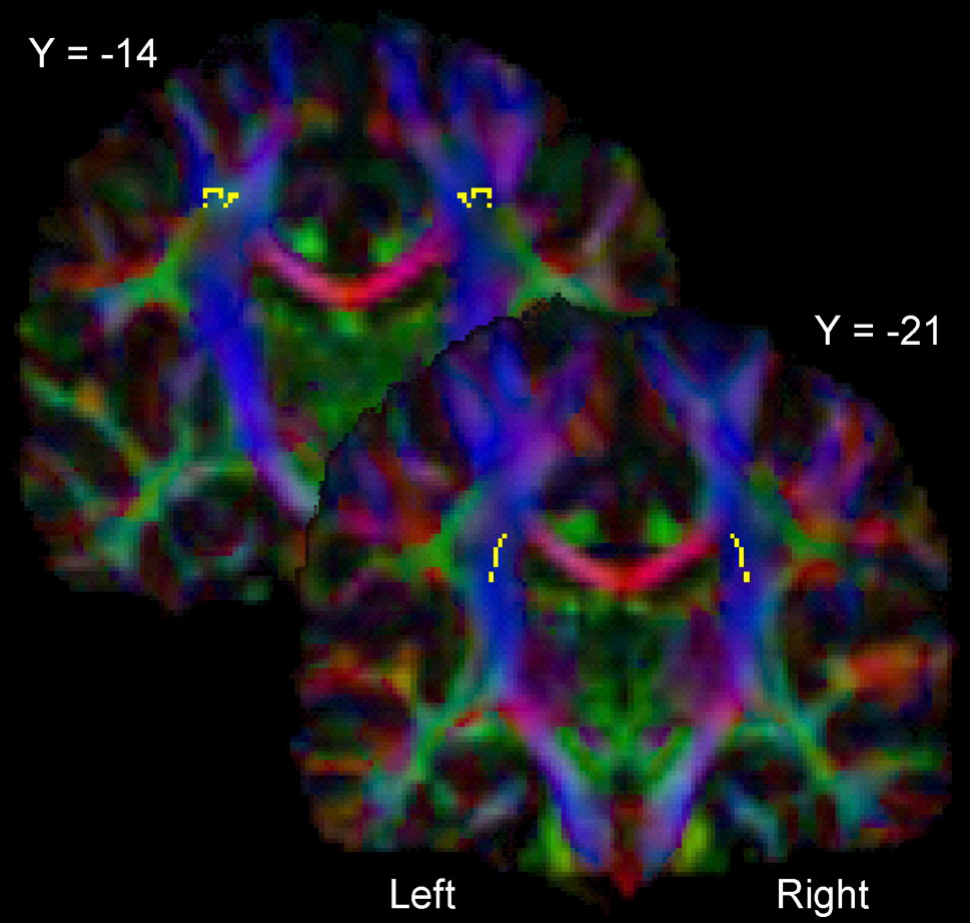
Statistical parametric maps showing those voxels where the *right*–*left* difference in FA shows a significant linear relationship with the right–left asymmetry in* circle* drawing as indexed by LI_CV_ (*p* < 0.05, correction for multiple comparisons). The voxels are presented as axial slice overlaid on the color-coded group FA map. The* color-coding* depicts the direction of the primary eigenvectors of the diffusion tensor in each voxel as follows: anterior-posterior in *green*, superior-inferior in *blue* and* left*–*right* in *red*. The two slices shown are MNI slice *y* = −21 (corona radiata) and MNI slice *y* = −14, depicting a complex region containing crossing fibers

We also performed a complementary voxel-wise analysis on the whole white matter skeleton. It revealed two significant clusters, displaying a linear relationship between left–right FA asymmetries and asymmetry in circle drawing (Fig. 6). Both clusters were located in the CST; one at the level of the superior corona radiata and the other one in about the same complex region as found in the slice-wise analysis. Both of them are positioned in our ROI and no voxels outside the CST reached significance after correction for multiple comparisons.

**Fig. 7:**
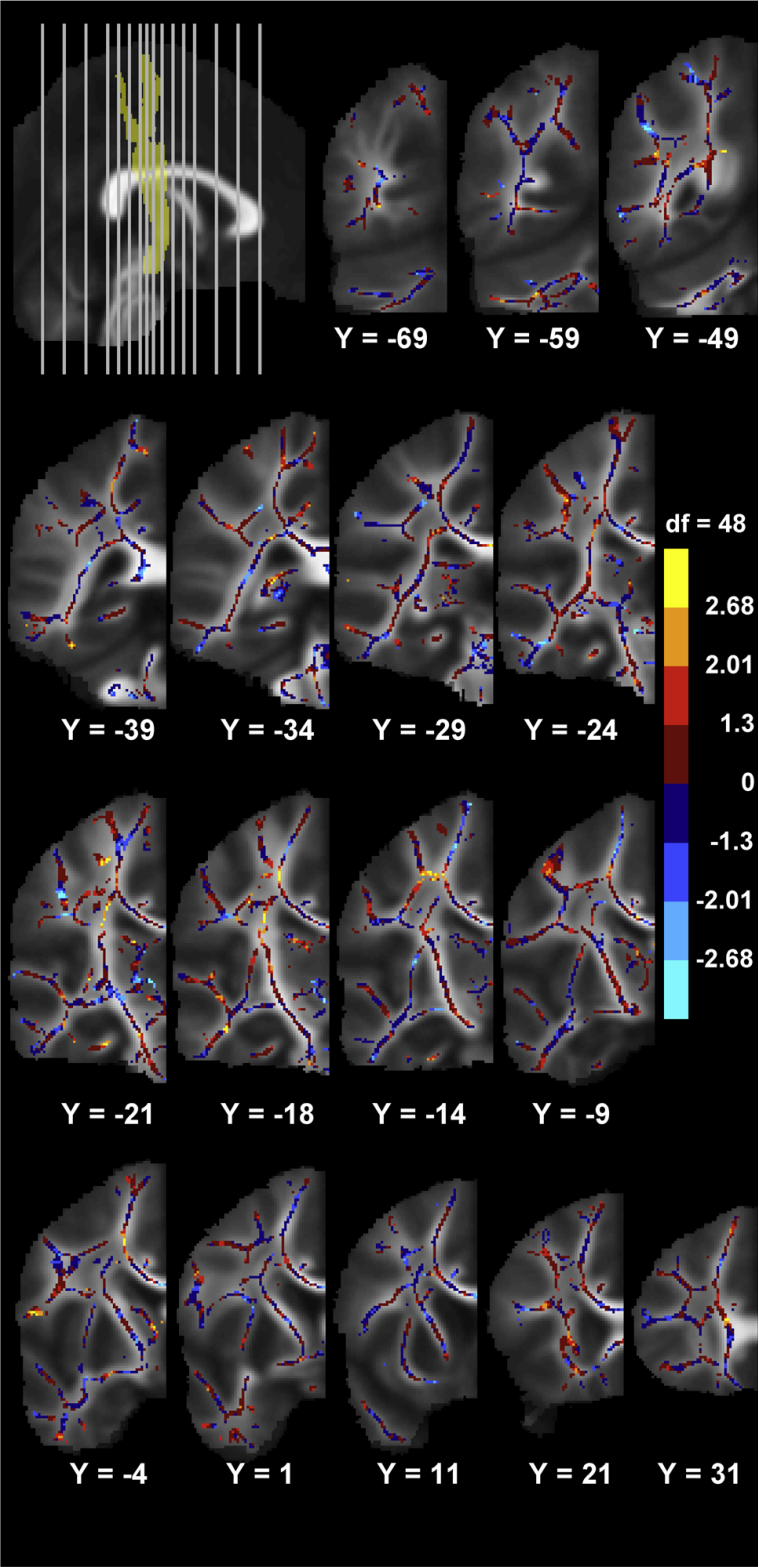
Effect-size map of the association between the *circle* drawing laterality index LI_CV_ and the right–left voxel-wise difference in FA-values, corrected for age and gender. The effect-size map is overlaid on the mean symmetric FA images. Only one hemisphere is visualized as a difference score is investigated. The association of higher right–left voxel-wise FA difference with better right-to-left circle drawing performance is shown in *warm colors*. Negative values are shown in *cool colors*. The *color bar* shows the color mapping of all skeleton voxels to the values of the uncorrected *t*-statistics (*t* = 0, *p* = 1; *t* = ± 1.229, *p* = 0.2; *t* = ± 2.01, *p* = 0.05; *t* = ± 2.682, *p* = 0.01). The MNI coordinates for the coronal images are given under each image. In the *upper left* corner the position of the coronal slices shown are depicted on the mid-sagittal image with the position of the CST schematized. Coronal slice −69 is displayed to the *far left* and 31 to the *far right* on the mid-sagittal image

Based on the whole skeleton analysis, we generated an effect-size map showing the regional distribution of the association between LI_CV_ and left–right FA asymmetry in the brain (Fig. 7). The symmetric TBSS-skeleton comprised a total of 56.308 voxels per hemisphere of which 1.713 (3.04 %) had a *t*-value above 2.0106 (*p* ≤ 0.05, uncorrected), while 1.278 (2.27 %) had a *t*-value below −2.0106 (*p* ≤ 0.05 uncorrected). This corresponds to 5.31 % of the total number of voxels in the skeleton which would be expected to occur by chance. Within the pre-defined CST ROI (1.785 voxels), this summed percentage was 9.47 % (169 voxels). In accordance with the main result based on left–right FA asymmetry of the entire CST, the vast majority of the significant voxels showed a positive linear relation between LI_CV_ and left–right FA asymmetry. 163 out of 1785 voxels (9.13 %) had a *t*-score >2.0106, whereas only six voxels (0.34 %) had a *t*-score < −2.0106.

## Discussion

This is to the best of our knowledge the first study linking a functional right–left asymmetry in manual skill to a microstructural right-left asymmetry of the corticospinal tract (CST) in the adolescent brain. In typically-developing adolescents, we combined fine-grained kinematic measurements to characterize the regularity of circle drawing movements and non-invasive brain DTI imaging to measure FA and MD in the CST. We found that higher FA values in the left relative to the right CST were associated with higher proficiency in right-hand circle drawing relative to left-hand circle drawing. The individual expression of left > right FA differences in CST predicted how much the right hand outperformed the left hand in terms of producing temporally stable velocity profiles in a simple circle drawing task. This link between right–left asymmetry in CST microstructure and circle drawing skill was not driven by inter-individual differences in preferred hand use, because all participants were consistent right-handers.

The microstructural properties of the right and left CST jointly contributed to the right-left difference in fluent circle drawing. When modeled simultaneously, FA in the right and left CST contributed significantly to the prediction of individual right-left differences in drawing performance, but not when modeled separately. In the simultaneous model, mean FA values in left CST showed a positive linear relationship with LI_CV_, whereas mean FA values in right CST showed a negative relation with LI_CV_. Our results are in line with those of a recent study in a small sample of 14 adults showing significant associations between manual dexterity in grip strength and finger-tapping performance and left higher than right FA asymmetry of respectively the CST and intrahemispheric connections between the pre- and post central gyrus (Rose et al. 2012). Together, these results strongly indicate that asymmetry indices based on a direct comparison of FA in corresponding white matter pathways of the left and right hemisphere, as compared to regional FA values in a single hemisphere, are better suited to identify structural correlates of lateralized function in the human brain. This notion is also supported by animal work showing the relevance of microstructural asymmetries between homologous areas for lateralized brain function (Ocklenburg and Güntürkün 2012).

The present study was designed to identify a microstructural correlate of right-left differences in manual ability (i.e., dexterity). We only included dextral adolescents with a clear preference for using the right hand during common daily manual tasks. While the focus on consistently dextral individuals minimized the confounding effects of preferential hand use, the present study provides no information about the relation between preferred hand use (i.e. handedness or dextrality) and microstructural asymmetry of the corticospinal tract. Indeed it would be very interesting to assess this function-structure relationship in left-handed individuals. Another open question is how much the degree of handedness modulates the relationship between the right–left asymmetry in manual skilfulness and the microstructural asymmetry in the corticospinal tract. These questions need to be addressed in future studies.

Using a different MRI-based microstructural marker, Hervé et al. (2009) studied the effects of handedness and dexterity on structural asymmetry of CS in a large sample of adolescents (*n* = 409), aged from 12 to 18 years. The CST appears relatively hypointense on *T*_1_-weighted MRIs which has been attributed to the presence of large corticospinal axons (Barkovich, 2000; Yagishita et al., 1994). Taking advantage of the relation between *T*_1_-weigted signal intensity of the CST and density of large-calibre CST axons, Hervé et al. (2009) performed a ROI analysis to compare “apparent grey matter density” (aGMd) in the putative CST in the posterior part of the internal capsule. They found a strong left > right hemispheric aGMd asymmetry in adolescents which was less pronounced in individuals with preferred left hand use (Hervé et al. 2009). In apparent contrast to our present results, left > right hemispheric aGMd asymmetry did not reflect inter-individual variations in manual proficiency as indexed by the Grooved Pegboard task (Hervé et al. 2009). Since the DTI-derived FA measure might reflect slightly different properties of the CST than the *T*_1_-derived aGMd measure, FA might be more sensitive than aGMd to reveal a relation between right–left asymmetry in CST microstructure and dexterity. The discrepancy might also be related to differences in the type of task used to determine asymmetry in dexterity. Our circle drawing task probed the temporal stability of muscular synergies of distal finger muscles, while the pegboard task used by Hervé et al. (2009) did probe a combined grasping-aiming skill involving distal (grasping) and proximal (aiming) synergies.

Taken together, the work by Herve et al. (2009) and the present study suggest that the microstructure of the CST in the adolescent brain is related to both preferred hand use and skill level, but different MRI-based microstructural markers might be more closely related to handedness or dexterity. The question how well various MRI measures of CST microstructure capture functional right–left asymmetries in relation to handedness or dexterity remains to be systematically studied in a multi-modal MRI study. Since the relationship between CST microstructure and motor hand function might change during brain maturation, a longitudinal study covering the transition from childhood to adulthood is warranted.

When comparing the microstructure of right and left CST, asymmetry in mean FA but not mean diffusivity predicted the right-left difference in fluent circle drawing. In other words, the directionality of regional water diffusion in the CST rather than its direction-independent magnitude of water diffusion reflected the right-left asymmetry in dexterity. FA values are considered to reflect axonal density, diameter, myelination and organization, and fiber coherence within a given voxel and are taken as an index of structural connectivity. A number of DTI studies have linked regional FA variation in white matter tracts with inter-individual performance differences in tasks probing specific cognitive abilities or skills (Wolbers et al. 2006; Madsen et al. 2010; Vestergaard et al. 2011; Madsen et al. 2011). Regional FA values in tracts linking different brain regions have been shown to be related to functional connectivity in a way that increased structural connectivity predicts increased functional connectivity (Damoiseaux and Greicius 2009; Groppa et al. 2012; Jung et al. 2012).

The regional FA value is determined by perpendicular (λ_⊥_) and parallel (λ_║_) diffusivity. Therefore, the relationship between right–left asymmetry in FA and circle drawing might have been caused by an asymmetry in λ_⊥_, λ_║_, or both. We found that λ_**║**_ in left CST was positively associated with right-left asymmetry in drawing performance (as indexed by LI_CV_), whereas λ_**║**_ in right CST showed a negative linear relationship with LI_CV_. Conversely, λ_⊥_ in left CST displayed a negative linear relationship with LI_CV_, whereas λ_⊥_ of the right CST showed a trend towards a positive linear relation with LI_CV_. We therefore conclude that DTI-based measures of perpendicular (λ_⊥_) and parallel (λ_║_) diffusivity jointly contributed to the asymmetry in mean FA between the left and right CST and hereby, to right-left differences in regular circle drawing. This raises the question how regional diffusion properties of water, as derived from DTI, relate to the histological features of the CST, such as axonal density, diameter and myelination, which determine its neurophysiological function (Hartline and Colman 2007). In a mouse model, increased regional *λ*_⊥_ values coincided with the extent of demyelination and were distinct from axonal damage (Song et al. 2005). In infants, it has been found that FA and *λ*_⊥_ but not *λ*_║_ are structural markers of myelination and that changes in these two measures are closely related to functional improvement in electrical conduction efficiency (Dubois et al. 2008). Specifically, increased electrical conduction speed is positively correlated with FA values and negatively correlated with λ_⊥_ values. As mentioned previously, the large and heavily myelinated corticospinal axons make monosynaptic connections with cervical motor neurons supplying the hand and are thought to be critical to manual dexterity (Lemon 1999, 2008). Therefore, it is tempting to speculate that a higher number of very large corticospinal axons along with increased myelination might cause higher FA values in the CST contralateral to the more proficient hand. Yet it is important to bear in mind that the neuroanatomical interpretation of DTI-based metrics is not straightforward. A number of other tissue properties such as axonal density, axonal diameter, extracellular volume fraction as well as crossing fibers and tract geometry determine water diffusion in addition to the degree of myelination and the range of axonal diameters in the CST (Gulani et al. 2001; Beaulieu 2002; Mukherjee et al. 2002; Hasan and Narayana 2006; Jito et al. 2008).

The main analysis was a ROI analysis based on the mean FA value of all voxels in the TBSS skeleton belonging to the CST. This ROI approach lacked topographic specificity within the CST. Yet we were interested to know whether the microstructural right-left asymmetry in FA was expressed homogeneously throughout the CST or whether the expression of microstructural asymmetry varied along the CST. To this end, we computed slice-wise effect size maps which consisted of axial slices along the CST (Fig. 5c). The slice-wise map pinpointed three sections of the CST where the right-left asymmetry in FA was particularly pronounced, including a voxel cluster in the internal capsule, superior corona radiata and a complex region containing fibers from different tracts, e.g. corpus callosum, CST and superior longitudinal fasciculus. The latter two clusters were linked to manual dexterity not only on slice level, but also on single-voxel level after correction for multiple comparisons. The spatially variable expression of right–left asymmetry in FA along the CST can at least partially be explained by the fact that many voxels that are assigned to the CST in the TBSS skeleton contain fibers from other tracts that cross the CST and hereby may attenuate the effect size of microstructural CST asymmetry. These tracts serve different functions and feature their own characteristics. In particular, tracts related to language functions exhibit asymmetry themselves (Vernooij et al. 2007; Friederici 2009), which is not related to motor proficiency. For example, the corona radiata comprises the corticospinal tract, the corticopontine tract and the corticobulbar tract. Callosal fibers and fibers of long sagittal fiber tracts blend with corticospinal axons in the respective voxels. Furthermore, the corticopontine tract and the superior thalamic radiation are situated close to each other and both tracts might influence the regional FA values. Within the CST, the corticospinal projections are not only targeting the hand. This implies that those voxels containing the corticospinal projections to the upper limb are likely to exhibit a stronger relation between right–left FA asymmetry and right–left asymmetry in dexterous hand use. Tractography could help to distinguish corticospinal projections originating from the motor hand area from other descending projections and clarify whether the link between structural asymmetry and manual proficiency is specifically expressed in the corticospinal fibers conveying the descending motor commands to the hand.

Our study had a cross-sectional design covering an age range from 11 to 16 years. We observed a general improvement in the temporal regularity of circle drawing for both, left and right hand. Accordingly, the relation between both hands in terms of motor performance remained unchanged and no significant age effect on right–left asymmetry was seen. With respect to DTI-based microstructural measures, we found a relative decrease in MD and increase in FA with age, which fits well with previous reports (Eluvathingal et al. 2007; Lebel et al. 2008; Madsen et al. 2011). With respect to right-left asymmetry, the relation between MD values in left and right CST was comparable across the age range, suggesting a symmetrical maturation. In contrast, FA values exhibited an age effect only in the left CST but not in the right CST. This divergence is in agreement with a previous study showing that age-dependent increases in FA-values may diverge for homologous tracts in the right and left hemisphere (Eluvathingal et al. 2007). However, a prospective longitudinal study is needed to properly clarify this issue. Here it would be interesting to start at pre-school age given the relatively protracted maturation of the CST during childhood and adolescence (Westlye et al. 2010) in order to obtain better insight into the developmental trajectories of manual proficiency.
